# Planning for Sustainable Green Urbanism: An Empirical Bottom-Up (Community-Led) Perspective on Green Infrastructure (GI) Indicators in Khyber Pakhtunkhwa (KP), Pakistan

**DOI:** 10.3390/ijerph191911844

**Published:** 2022-09-20

**Authors:** Muhammad Rayan, Dietwald Gruehn, Umer Khayyam

**Affiliations:** 1 Research Group Landscape Ecology and Landscape Planning (LLP), Department of Spatial Planning, TU Dortmund University, 44227 Dortmund, Germany; 2 Department of Development Studies, School of Social Sciences and Humanities (S^3^H), National University of Sciences and Technology (NUST), Islamabad 44000, Pakistan

**Keywords:** climate change, adaptation, urban green infrastructure, community participation, KP, Pakistan

## Abstract

Rising vulnerability of the urban green infrastructure (UGI) is grabbing global attention, for which inclusive urban landscape and greening policies (ULGP) and frameworks are crucial to support green growth. As such, this research intends to explore the local community’s perspective to assemble sustainable UGI indicators for vital taxonomy of the urban green space (UGS) elements, aiming to develop a multi-functional and sustainable UGI-indicator-based framework that is eco-friendly and supports green-resilient cities in Khyber Pakhtunkhwa (KP) province, Pakistan. An in-depth household survey was executed in three KP districts: Charsadda, Peshawar, and Mardan, placing self-administered 192 questionnaires while covering themes around climate change adaptation, urban resilience, and UGI. Relative importance index (RII) and the interquartile range (IQR) methods were set up for data analysis that revealed excellent reliability (α > 0.88) and internal consistency. The results confirmed community-based UGI indicators with a focus on promoting green-energy-saving strategies as e-imp (level 9, RII = 0.915), while other (ten) UGI indicators as important (RII = 0.811–0.894) and (eleven) as moderately important (RII = 0.738–0.792). These UGI indicators were found to be enhanced by UGS elements (RII ≥ 0.70). These findings provide a foundation for urban policy change and the development of a sustainable UGI framework to build an eco-regional paradigm for greener growth.

## 1. Introduction

Urbanization leads to the shrinkage of urban green spaces, which directly contributes to extreme climatic hazards such as flooding, drought, urban heat island effect, etc. These hazards then further result in the degradation of ecosystem functions (ESF) and the loss of biodiversity and affect human health/well-being [1,2,3,4,5]. The experts anticipate that the climate change observed today and in the foreseeable future will be influenced by the variability of anthropogenic forcing [6]. If we cannot limit global climate change, there will be far-reaching repercussions on nature and society [7]. The global vulnerability of cities to climate-related hazards and stresses is expected to increase due to an increased built-up footprint compared to the population growth rates. According to research, it is estimated that the urban population will grow by 72% from 2000 to 2030, while the built-up area of cities (with 100,000 residents) will grow by 175 percent [8]. The incremental trend of the world population and anthropogenic activities has changed the land cover and contributed to the greying of the natural landscape. These harmful impacts of urbanization and the corresponding high pressure on the natural environment, at an unprecedented rate, are badly hampering urban growth in the major cities of Pakistan. In Pakistan, the urbanization rate is amplified from 32.98% (year 2000) to 36.91% (2019), with further projections to reach 50% by 2025 [9]. This upsurge is transforming the local attitude towards green spaces; thus, urban centers receiving less consideration become unsafe [10] and evolve multidisciplinary climatic challenges. Amongst other challenges, ‘urban flooding’ remains the most threatening climatic hazard with the power to endanger human safety and frighten natural resources and ecosystems. Furthermore, jeopardizing the socio-economic fabric of urban (flood-affected) inhabitants stays common. 

These issues call for urban green infrastructure (UGI) (UGI planning is defined as “a network composed of open spaces, waterways, gardens, forests, green corridors, trees on streets, and open spaces, bringing many social, Economic and ecological benefits” [11]. Another UGI version exists as “an interconnected network of natural areas and other open spaces that conserves natural ecosystem values and functions sustains clean air and water and provides a wide array of benefits to people and wildlife” [12]) to enhance urban sustainability [13]. UGI is perceived as a nature-based and cost-effective solution to achieve resilience in the land use planning process to mitigate the ever-rising climate uncertainties [14,15], a revamp of all existent contemporary ideas concerning green space planning [16,17], an approach to enrich the health of the ecosystem, minimizes the surface-water runoff, improves water infiltration rate [18] and a cost-effective strategy to mitigate urban floods. Thus, UGI planning is already testified and declared important in countries such as Germany, the UK, and the Netherlands, where it is encouraged to promote innovative nature-based green solutions for climate change mitigation and adaptation [19,20,21,22]. Hence, it is confirmed that planning instruments (such as UGI) play an imperative role in minimizing the urban flooding effects, thereby enhancing the socio-ecological well-being of any region. Based on its strengths, this nature-based green (NBGI) approach stands as an applicable instrument for sustainable climate-risk management (SCRM) in cities [23], yet importantly, in bitterly climate-affected countries such as Pakistan.

### 1.1. Establishing a Niche: Climate Change Impacts

Pakistani urban areas pose multifaceted climatic encounters [24]. The consistent urban flooding events observed in recent years are putting lives and livelihoods at stake [25]. It is these growing incidences of floods, strong monsoon circulation, surface temperature rises, etc., that are making the country highly vulnerable and positing it (as per the climate risk index-CRI) (CRI is research that is centered on a comprehensive and accurate database of climate hazard effects observed in all countries in the world. In addition, low-income countries need to utilize the index as a warning signal to equip themself completely for future catastrophic disasters. www.germanwatch.org/en/cri, (accessed on 11 June 2021)) the eighth most vulnerable country to climate hazards [26]. Therefore, the disastrous impacts on ecosystems, biodiversity, agriculture, human settlements, human health, etc., are profound with different levels of adaptability [27,28]. Such devastating disasters for a country such as Pakistan (an agrarian economy) directly hamper the agricultural sector, which contributes 21.9% of GDP and employs 45% of local labor [29,30]. These disturbing lives and livelihoods remain prominent in the major/mega cities, which is further linked to massive and unplanned settlements at the expense of decreasing forest cover [4,31]. Other factors contributing to this issue are high population density, building of new colonies or expansion of physical infrastructure, etc., that are removing the green cover and urban green-spaces and rising air pollution [32,33]. These aforementioned problems prevail due to non-existent UGI planning in the existing urban plans and policies of the country, where such effective strategies are perceived only as a luxury urban activity. It is mainly associated with beautification (though not an essential urban amenity) to influence urban resilience against climatic hazards [10,34,35]. 

The whole alarming situation is linked to the regional non-resilient outlook toward unbalanced and reactive urban planning policies [36] that leads to unplanned settlements–further enlarging the environmental issue in the country [37]. Aside from the planning deficiencies (as outlined above), other contributing factors are inadequate ULGP, weak laws and enforcement, un-due influence, lack of scientific knowledge, lack of awareness, non-existence of PP is recognized as the effective tool to promote community stewardship in the planning and decision-making process to bolster nature-based-green infrastructure (NBGI) initiatives in land-use planning; effectively tackling socio-environmental problems at grassroots levels [38,39,40,41,42] approach, etc., all contributing to the transformation of green-spaces into urban functions/activities [10,35,43]. This exerts constant pressure on land cover, so deterioration of UGS elements. These issues declare Pakistani mega cities highly vulnerable to natural calamities, with no exception of the northwest territories of KP [44].

**Figure 1:**
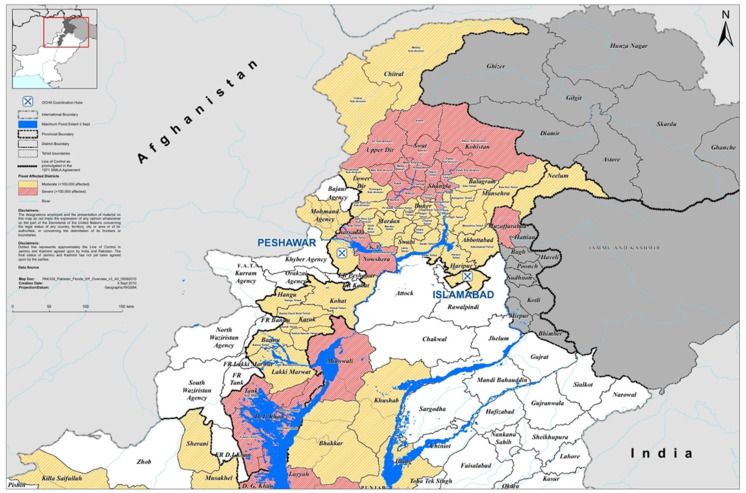
Flood-affected districts of KP province. Map Source: [24].

At a national level in Pakistan, KP province suffered predominantly from consistent flooding events in the last decade [25], which marks this area as highly vulnerable and risky to in-daunting events, accounting for massive economic and human losses [22,36,45] Generally, these damages are linked to region geographic position and topographical features. The area lies on the bank of Swat Kabul, Kunhar, and Panjkora rivers’ basin that originates from the high mountains of Hindukush, Himalayas, and Karakoram ranges. Being at water banks, it enhances the catchment area’s vulnerabilities to urban flooding (Figure 1). In addition, the issue also stresses the built environment service, which leads to the over-exploitation of natural green barriers [10], thereby endangering the urban ESF and human health/well-being in urban settlements [46,47,48]. Therefore, tackling the underlying causes and destructive effects of climate change in this region requires an immediate effort to examine the nexus between the UGI and climate-resilience notions to be incorporated (holistically) in the land-use planning process [26,27,28,29]. It is to develop a rich, multi-functional/inclusive/sustainable UGI-indicator-based (framework) model structured according to the local built environment. Such a model should be grounded on the (native) community perspective or the PP process—that further leads to strengthening the climate-resilient strategies, green spaces (GS), ecosystem functions (ESF), and human well-being in catchment areas. 

### 1.2. PP for UGI and UGS

PP (as an effective tool) can facilitate community stewardship in the planning and decision-making process, though so far less considered within the Pakistan planning context [11,12,30,31,32,33,34,49,50]. Consequently, PP deems more effective in understanding the complexity of interactions among the ecosystems and humans [38,51,52]. Hence, PP facilitates in drawing ‘human-nature’ studies/concepts for not only finding nature-based green infrastructure (NBGI) solutions [53] but, in turn, enabling human societies to enhance their adaptative capacity and build resilience against (ever-rising) environmental hazards, e.g., urban flooding [44,45]. To further add, a (bottom-up) PP approach help for a successful transition to green action plans (GAP) [38].

It is established that there is a dearth of research studies in Pakistan towards developing theoretical and empirical foundations for UGI planning and implementation, which is a prerequisite for an eco-friendly and climate resilience environment in the country (in general) and in KP (in particular). Though planning authorities in Pakistan adopt spatial technologies in-order to develop land-use maps of major urban districts, these interventions are still in their infancy [54,55] and usually require more time and financial resources [34,56], especially in the non-collaborative and unilateralism environment (with undue influence) that Pakistan possesses [43,49].

Hence, to bridge this gap, this (novel) research study intends to develop a rich body of multi-functional conceptual UGI-indicator-based framework/model, which can be grounded upon “Triple Bottom Line” (TBL) (The triple bottom line (TBL) refers to sustainability’s environmental, socio-cultural, and economic dimensions. It is the most commonly accepted model used in most urban sustainability applications [46,47,57]) sustainability and adapted to the local context. Such a potential framework encompasses a set of core sustainable UGI indicators and vital taxonomy of UGS elements. To bottom-up oriented framework/model testify and validate the margin between essential and inessential potential UGI indicators and green space elements. Here, the local community evaluates the significance of the sustainable UGI indicators and their relationship with multiple UGS elements, according to the native built-in environment. It is because (i) the effectiveness of UGS structures (usually) depends on the spatial contextual factors (socio-cultural and economic) of any region where they are examined [15,50,58,59,60] and (ii) not all the UGS elements had an excellent functional linkage to improve the resilience of respective sustainable UGI indicators while coping with the gradual climate change.

In this sense, this research study is inception that breeds the PP approach to develop an inclusive, sustainable UGI-indicator-based framework to build green and resilient cities in the KP province. It is also imperative that this UGI model, developed through this study, can be adapted to the native spatial environment-will provide a proactive and long-term way for ULGP and guidelines for CC mitigation/adaptation. This may lead to a well-balanced relationship between anthropocentrism and eco-centrism activities for KP and beyond. Moreover, such a framework will lead to encouraging innovative green grass-root initiatives, with the mandate to build a new eco-cultural paradigm to enhance the adaptive capacity in sustained human settlements. This will inevitably open up a new domain of study to gradually probe more deeply into innovative community PP approaches when planning nature-based green adaptation techniques for climate change adaptation.

### 1.3. Study Aim and Research Questions

This research study aims to analyze the community perspective (through the bottom-up PP approach) to gauge the locals’ insightful view regarding UGI-indicator-based framework/model. It is to obtain a greater consensus among the local community to find a relationship between sustainable UGI indicators and (potential) taxonomy of UGS elements, as per the native built environment. This then leads to validating the sustainable UGI framework/model, which fits best in the local socio-economic and cultural context. Such an effort would contribute to enhancing green-spaces, besides alleviating vulnerability towards climate hazards. They also improve regional socio-ecological resilience. It also builds climate-resilient cities in KP territory under a community participation approach–promoting a sense of community ownership. Therefore, the study intends to find answers to the following three research questions:

i. What is the level of the local community’s understanding of Climate Change and UGI?
ii. Which essential UGS elements strengthen the resilience of (sustainable) UGI indicators?
iii. What type of UGI-indicator-based model contributes to building a green climate-resilient city-state?

## 2. Research Methodology

**Figure A1:**
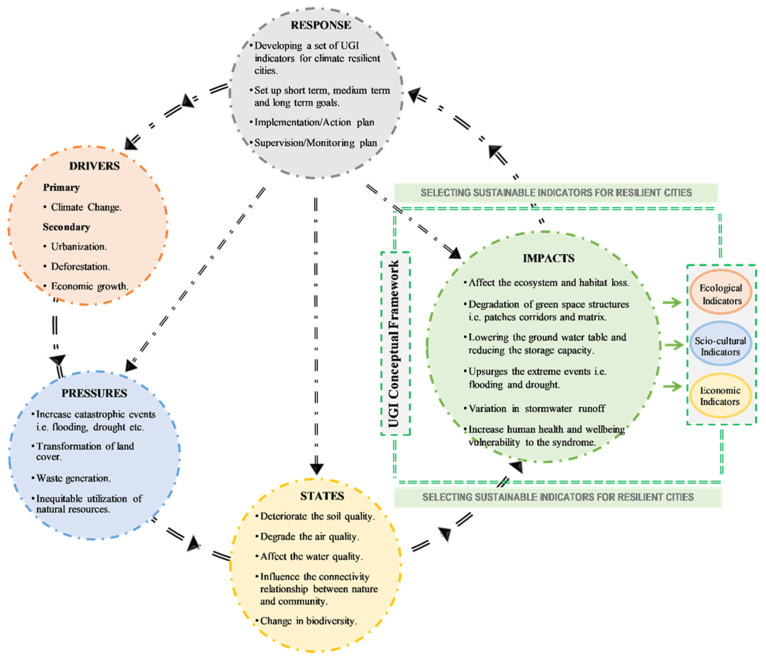
Relationship among the anthropogenic activities and UGI for resilient cities. Source: [48].

**Figure A2:**
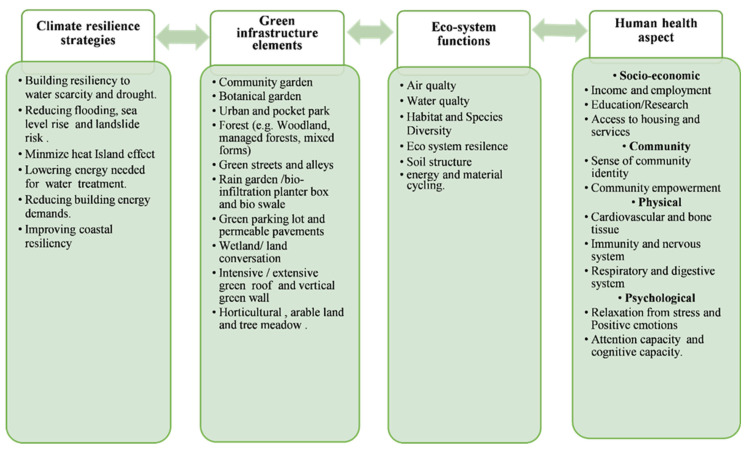
Conceptual base model: climate resilience strategies, ecosystem functions, human well-being, and GI elements. Source: [48].

This research has adopted the UGI (conceptual base) framework model developed by the author [48], grounded on two conceptual frameworks: (i) Driver pressure state impact response (DPSIR) framework that aims to conceptualize the relationship between UGI elements and anthropogenic activities to build climate-resilient cities and (ii) incorporation of the model, proposed by [14], which is further enhanced by inserting three additional components, (a) climate resilience strategies, (b) eco-system function—ESF and then (c) the UGI elements as suggested by [15,17,59,61], but revised to build a strong correlation among them [48] (for details, see Appendix A: Figure A1 and Figure A2). Additionally, semi-structured discussions with multi-stakeholder (planning experts and community) in Pakistan were conducted regarding the potential role of NBGI initiatives’ in promoting an eco-friendly and climate-resilient environment, resulting in nine cross-cutting themes [34] (see Appendix B: Table A1). 

The consolidated integration of both models and concepts intends to build a cohesive, sustainable UGI framework. This framework is perceived to enhance urban resilience against (ever-rising) environmental hazards and (simultaneously) minimize the degradation of the urban ecosystem health. These conceptual models and new cross-cutting themes (an innovation of this study) are regarding the potential role of UGS infrastructure in addressing SCRM. This, in-tern, assists in determining the (potential twenty-two, some placed under main headings) UGI indicators that were classified into three main sustainability categories (i.e., ecological, socio-cultural, economical). It was conducted along with the ten (community garden (CG); botanical garden (BG); urban park (UP); forest (FO); green streets (GR); rain garden and bio-swale (RG); green and permeable parking area (GPA); wetland (WL); green roof and green wall (GRW); and horticulture (HO)) vital UGS (quantitative) elements to accomplish the research questions. Of course, there could be other indicators, such as institutional and political. However, that is out of the scope of this research and can be considered in future research. The potential UGI indicators and green elements are mainly quantitative, and the relative importance index (RII) and interquartile range (IQR) analysis technique employed applied to calculate the relative significance of each sustainable UGI indicator, as well as the UGS elements, as analyzed by the local community (within the real-life context). This method is recognized best approach for ordinal-scale surveys [62,63,64,65].

### 2.1. Study Area, Sampling Technique, and Survey Design 

**Figure 2:**
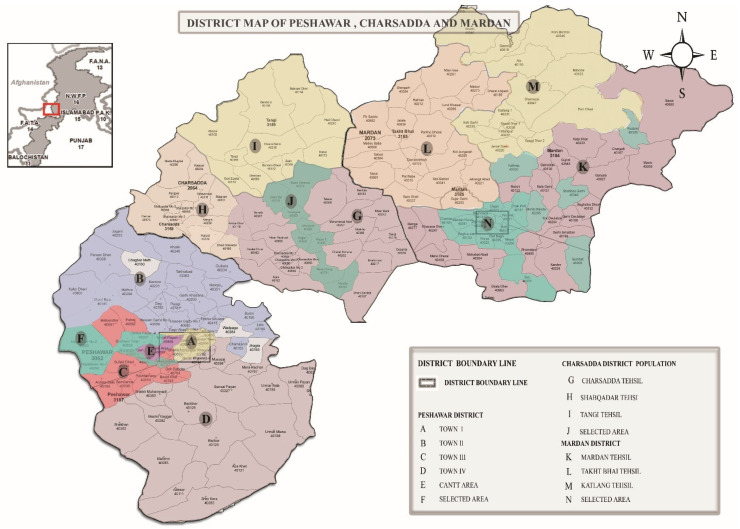
District, tehsil, and union council map of Mardan, Charsadda, and Peshawar. Source: Authors’ compilation from [24,66].

Multi-stage sampling technique is used. Firstly, selection of the municipalities (Tehsil) (Based on the higher population, the municipalities (Tehsil) in each district are selected (Table 1)) in each study district (Peshawar, Mardan, and Charsadda) (Table 1), and secondly; the selection of sub-municipalities (Union Council-UC) in each tehsil in the KP province, which is based on population census datasets [66]. (For determining the UC, this research integrated the interquartile range (IQR) technique with criteria 1. IQR is an efficient method for determining cut-off points [64,67,68,69,70]; based on population census datasets [66] and above the cut-off point (mid-point), all UCs were selected for the field survey (Figure 2). This methodology was adopted as there is no official list of the residential houses affected by climatic uncertainty within UCs exist. Moreover, each municipality has a minimum of 20 and a maximum of 37 sub-municipalities (called Union Councils-UCs). So, the most floods affected, time (cost) efficient areas, and safer strategies (in time of COVID-19 pandemic) were selected for study purposes). Thirdly: the flood-affected Households (HHs) were consulted in the community survey (Table 2 and Figure 2) executed in the case study area from October 2020 to mid-December 2020. 

In total, 192 HHs [with 95%confidence level (CI), ±5% margin of error (MoE)] in which 64 HHs belong to Mardan, 57 HHs to Charsadda, and 71 HHs from Peshawar tehsils. A total of 1198.8 sample population (community) were consulted to study the subject trends, as per Cochran (1977) (Table 2), succeeded over from pilot testing to check the independence of various indicators and necessary modification in the questionnaire, as per the inputs (from local govt. officials, two expert consultants, three academicians, and three community members), which were conducted to check its feasibility, inclusiveness, and precision. This approach helped to do prior minor amendments (see Appendix C) questions’ appropriateness and time efficiency [73,74]. In general, the acknowledgment level remained acceptable for generalizing the results over the whole study sample population [75,76]. 

**Figure 3:**
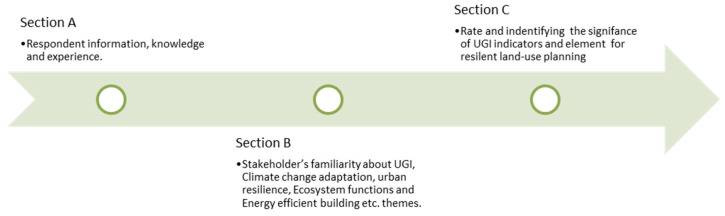
Community HH survey strategy. Source: [34].

The community-based empirical study employed the snowball technique in the case study to identify specific HHs, which served as a reference benchmark, selecting every fourth HH from the reference point as an HHs sample to obtain field data. This methodology was deployed since no official lists of flood-affected residential houses exist within UCs. To collect the study data, a structured survey questionnaire was designed with three Sections A–C. (“Section A was labelled as the demographic information, aiming to validate the respondent profile, knowledge, and location. The diverse category “trans” has been included as a third gender since the state approved it. Part B comprehends 4 questionnaires designed to verify the native community’s views on the potential definitions of climate change (CC), adaptation to CC, urban resilience, and UGI, as explained in Appendix D; Part C was divided into three sub-parts (environmental, socio-cultural, and economic), each comprising several queries to define and determine the significance of each UGS element and its relationship with sustainable UGI indicators. The Potential UGI indicators and UGS elements were developed by the authors in preceding research studies” [48]. “This process resulted in selecting the vital taxonomy of the UGS elements that enhanced the quality standard and health of respective UGI indicators and would build the urban interface in the KP region that is resilient against constantly rising environmental threats such as urban flooding” [34]), which consist of closed and open-ended questions (Figure 3). A Likert-scale approach was adopted [77,78,79] to register the participants’ responses so that the native community perspective of (potential and sustainable) UGI indicators and their relationship with multiple vital taxonomy green elements in the local urban environment can be easily explored. 

The demographic characteristic affirmed 65.6% as male, 22.4% as female, 12% preferred not to mention their gender, and no participant was from the third-gender category; this option was provided as the government of Pakistan officially recognizes “trans” as a third gender [80,81,82]. The participation percentage of Masculine is high compared to feminine participation because most of the KP region’s households are mainly male-headed [66]. Moreover, the other reasons behind this relatively low no are (i) the female HHs are very low, and the majority of HH are male-headed in the area; (ii) local social and cultural norms are challenging to gain access to females; (iii) society does not allow outsider males (authors) to directly interact with females in their areas—yet this study tried to include the maximum possible female as HH heads through volunteer enumerators understanding the local culture and knowledge of the study. 

Regarding the participants’ educational background, 73.4% had tertiary/higher education levels, 19.3% were intermediate, and 7.3% had secondary education (Table 3). The study also signifies nearly all representatives from the major four age groups had participated in the survey that shows the engagement of community individuals from all age groups. The age group with the highest frequency was 30–40 years (43.8%), followed by 34.4% in the age bracket of 20–30 years (The socio-demographics presented here only project all segments of the society, across all age groups, gender categories, and income and education levels were duly consulted. Their separate relationship for the study variables was neither intended to be covered nor they have influenced the broader findings of this study) (Table 3). The participants came from an array of socioeconomic backgrounds.

### 2.2. Data Analysis and Survey Reliability

**Figure 4:**
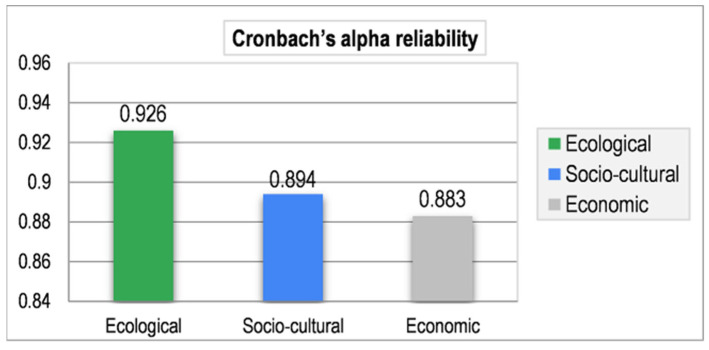
Cronbach’s alpha reliability. Source: Authors’ calculation using field data.

Data were examined through Microsoft Excel. Sections B and C (as of Figure 3) of the survey questionnaire used a question-based coding algorithm to segregate and examine the community responses. Cronbach’s alpha (α) was executed, and α-values (>0.7) confirmed data reliability (Figure 4).

As the proposed UGI indicators and UGS elements are quantitative, therefore the relative importance index (RII) technique was employed to examine community responses to build a composite (potential) UGI framework/model. It is to determine community satisfaction of UGS elements and UGI indicators, according to the local built-in environment, as executed in similar research studies for ordinal-scale surveys [62,63,64,65,83]. 

Based upon RII (outcomes), the importance level of each UGI indicator and UGS element was calculated. To ensure a rational quantity of UGI indicators and vital UGS elements (for respective UGI indicators from RII), two interrelated strategies were introduced: (a) adaptation importance scale criterion, as proposed by Chinyio (1998) [62] and Akadiri (2011) [83], yet inserting ‘four new levels’, so accounting for (in-total) nine-point importance levels [34] (Table 4). The scale ranges from “extremely unimportant” to “extremely important”, utilizing both Positive and negative weights (Table 5). The values substituted into formula 1 (Table 5) are from Table 6. It is to find variance in the significance levels amongst the UGI indicators and UGs elements because not all the GS elements positively enhanced the efficacy of the sustainable UGI indicators.

The second methodology was the adaptation of the Interquartile Range (IQR) technique to identify a specific cut-off point in the RII Values (RIV) of UGS elements. The IQR is a viable and effective technique to determine the difference between the median of the lower (Q3) and upper (Q1) quartile of the RII data set [64,68,69,70]. It also enables the identification of a vital (manageable) number of UGS elements for the respective UGI indicators according to the native spatial environment. The value of 0.70 is considered a cut-off point, which assists in determining the key UGS elements for each specific UGI indicator. This enhances the urban system’s ability to withstand climate threats of anthropogenic changes. The cut-off-point (RI = 0.7) is based on the Likert scale (Table 4), ranging from Moderately important to Extremely important. The cut-off point ((RI < 0.7) implies that not all the individual UGS elements had an excellent functional connection with the (respective) UGI indicators to fight climate change in the study areas.

## 3. Results and Findings

The results of the community-based (bottom-up) empirical field study were elucidated, firstly, the answer to RQ1: community’s understanding of climate change, adaptation to climate change, urban resilience, and urban green infrastructure themes. The next sections tackle (RQ2 and RQ3), determining key UGS elements that strengthen sustainable UGI indicators, thereby contributing to a (probable) UGI framework/model. The attempt to develop an essential resilience against the environmental challenges in the native built-in context.

### 3.1. Section A: Understanding the Local Perspective

**Figure 5:**
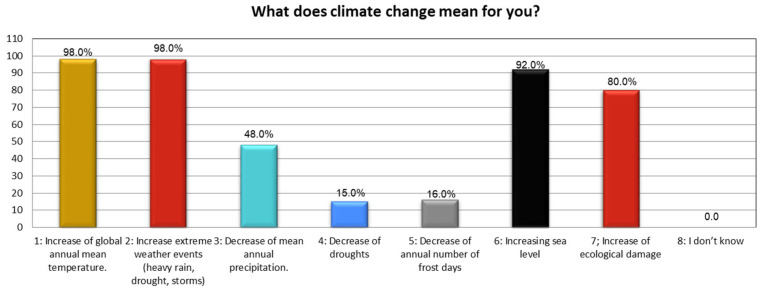
Defining climate change (CC). Source: Authors’ calculation using field data.

**Figure 6:**
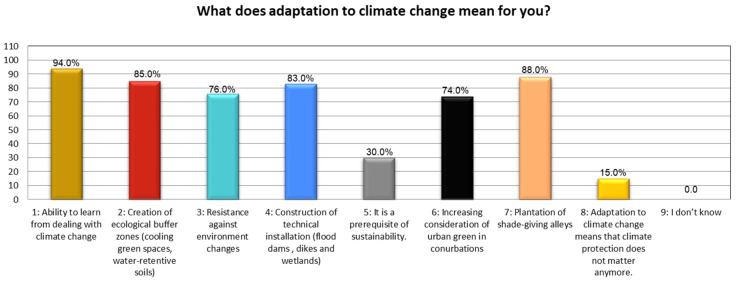
Defining climate change adaptation. Source: Authors’ calculation using field data.

The results represented that options one, two, six, and seven are more effective than other options, which achieve a lower percentage. The statistical investigation represents that more than three-fourths of the community believe that an increase in the global annual mean temperature poses severe weather events such as rising sea levels and, thereupon, damages the ecological health of both the urban as well as rural areas (Figure 5). This leads to endangering human health/well-being safety and destruction to urban eco-system functions in the study districts. Further results about “adaptation to climate change” in the native/local context found that options: one, two, three, four, and seven remained extremely important–receiving more than 75% Satisfaction Approval Vote (SAV) (Figure 6). This set of five important variables helps to outline the imperative concept of “adaptation to climate change (CC)” in an indigenous context, besides contributing to strengthening local adaptive capacity. They also assist in building resilience towards environmental hazards (i.e., urban flooding).

**Figure 7:**
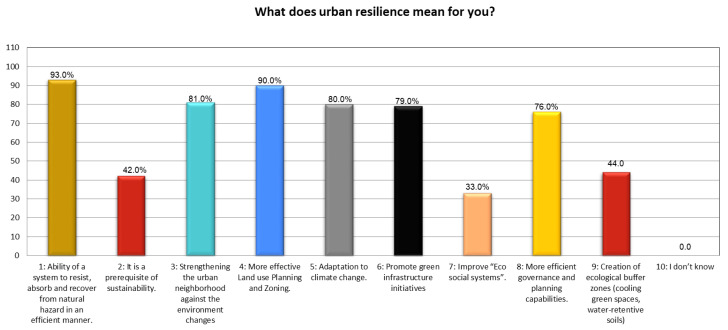
Defining urban resilience. Source: Authors’ calculation using field data.

**Figure 8:**
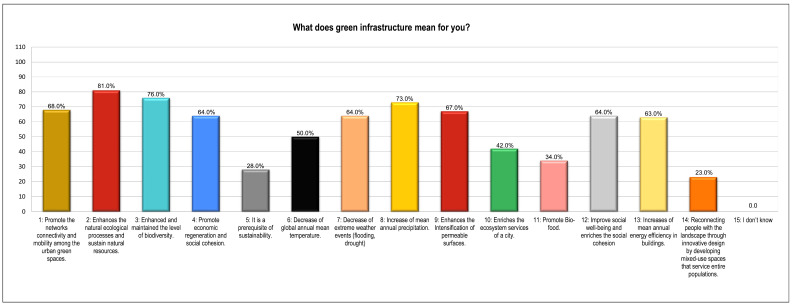
Elucidating urban green infrastructure (UGI). Source: Authors’ calculation using field data.

Furthermore, the results emphasize that the local community acknowledged ¾ of the potential options that are perceived as influential in gauging/defining urban resilience in the local context. The statistical result shows that options: one, three, four, five, six, and eight are more effective as they received >75% SAV than options two, seven, and nine (SAV < 50%) (Figure 7). This illustrated participants’ understanding and level of confidence in the potential alternatives, which may lead to green-growth approaches and a sustainable urban environment in KP. The results further elucidate the perception of the native respondents on the potential possibilities of UGI, so it is worth noting that option 2 is extremely significant compared to option 14, which received 81% and 23% confidence votes from the participants. Besides that, the community also endorses options: 1, 3, 4, 7, 8, 9, 12, and 13, though the confidence level varies between 60% and 70% (Figure 8). This certifies all the nine potential possibilities were viewed as a key standard to define UGI at the grass-root level in the study areas.

The results so far illustrate the community’s perspective and level of confidence in each potential possibility. This endorses multiple optimal approaches that can help to define the imperative themes such as climate change and adaptation, urban resilience, and UGI. Furthermore, such efforts enhance the inhabitant’s knowledge, beliefs, attitudes, and preferences regarding the potential role of NBGI in times of climate change. These investigations have the potential to contribute to the development of a sustainable UGI framework so as to build a green and climate-resilient city-state at the local, regional, and national levels in Pakistan.

The first part of the results concludes that most of the local community believes that its the increase in the global annual mean temperature that is resulting in severe weather events (e.g., storms, droughts, heavy precipitation, and sea-level rise) and disturbing the ecological system of the region. These situations are leading to risking human health and well-being, besides damaging urban green-space structures (quantity and quality), affecting ESF, and loss of biodiversity at all spatial levels. Therefore, the local inhabitants highlighted the need to create green buffer zones (such as wetlands, water-absorbent forest landscapes, etc.), construction of walls (at coasts), and more multi-scale plantations; only then can we ensure and enhance the adaptative capacity of the urban systems against the anticipated environmental challenges and recover from the natural calamities. There is a dire need to learn from the day-to-day experiences to deal with climate change, besides community-level adaptation to climate change. Additionally, there is a need for effective spatial land use and zoning plans to foster the rational use of urban land to develop various urban functions sustainably. This enables the transformation of the KP regions into eco-friendly and green climate-resilient city-state.

### 3.2. Section B: RII of Sustainable Indicators and UGS Elements for UGI Indicators

The empirical results of this section constitute answers to the study’s RQ2 and RQ3. Hence, this section is grouped into two sets to (i) investigation of key UGS elements that fortify the significance of each (sustainable) UGI indicator, as categorized in the TBL; (ii) identifying key UGS that play a fundamental function in enhancing the quality of UGI indicators ultimately contributes to a (probable) UGI framework/model that has the strength of encountering climatic hazards in urban settings.

#### 3.2.1. Determine the RII of Sustainable UGI Indicators

The potential UGI indicators were categorized according to their weights by using the RII formula. An example of the RII calculation is explained in Table 5 (the values substituted into the formula are from Table 6). Then, the RII technique was deployed to gauge the significance of all sustainable UGI indicators as per local community perspectives (Table 6). The below empirical evidence showed that all the UGI sustainable indicators were divided into three groups: moderately important (m-imp), important (imp), and extremely important (e-imp). One UGI indicator (promoting green energy-saving strategies) had achieved the (e-imp) level 9 with an agreed share of 0.915 RII values. Along with this, ten UGI indicators (imp) and eleven indicators (m-imp) were recognized with an RII value ranging from 0.811 to 0.894 and from 0.738 to 0.792, respectively (Table 6). None of the values was found between 1 and 6, i.e., from extremely unimportant to slightly important. Overall, the result has established that most of the UGI indicators fall into the categories of important (imp) and moderately important (m-imp), whereas only one ecological indicator belongs to the extremely important group. This portrays the indicator’s importance and distinctive quality based on the insightful review of the local community towards each UGI indicator. Such an effort would contribute to promoting sustainable green growth and, therefore, adds to building climate-resilient cities.

It is also important to note that if this research outcome is compared with the author’s previous (top-down) local planning expert studies [34,51], it illustrates that the importance levels assigned by planning experts to potential sustainable UGI indicators vary. For example, in the expert study, the ecological indicators received a higher acceptance level than the other two categories, but vice versa in the community-based empirical study.

#### 3.2.2. (a) RII Values of UGS Elements with Regards to UGI Indicators

The results have further determined UGS elements that improve the quality of each UGI indicator in making cities inclusive, eco-friendly, and resilient against environmental change. RII data set ranged between 0.37 to 0.95 values (Table 7). This reflects that, while dealing with climate-related disasters, not all the UGS elements assist positively in enhancing the efficacy of the UGI indicator. Only the UGS elements with an RII > 0.70 (Table 8) have the potential to enhance the efficacy of the UGI indicators against anticipated environmental challenges in the local urban context. These correlations enable to build of an inclusive and sustainable UGI indicator framework, besides supporting the formulation of green-growth strategies in land-use planning. Additionally, highlighting such vital taxonomy of green-space elements can improve the native community’s understanding of NBGI for climate change mitigation/adaptation. Thus, the community-based green strategies can help to build an eco-sustainable and climate-resilient environment in the urban interface of the KP province and beyond.

#### 3.2.3. (b) Identifying the Key UGS Elements

The finding further confirmed a functional linkage of each green element with the sustainable indicator, based on IQR values (0.60–0.76) with a cut-point of 0.70 (Table 8). These results highlighted important UGS elements for each UGI indicator, conditioned to local environments and community understanding. Overall, the outcome represents a pattern of variance, which signifies that each potential UGS element is characterized by a distinctive quality, and it does not improve the functional linkage and health of every UGI indicator. It is probably due to regional spatial context. Yet, the determined key green elements (Table 8) that perform a pivotal role in strengthening the resilience of respective UGI indicators also help to mitigate/adapt against the climatic variabilities in the urban settings.

It is also established that the city/urban appeal can be improved through various taxonomy of mixed-use green-spaces. It can be achieved more explicitly through the recommended UGS categories CG: “community garden”; BG: “botanical garden”; UP: “urban park”; FO: “forest”; RG: “rain garden and bio-swale”; WL: “wetland”; GRW: “green roofs and walls” and HO: “horticultural” [34]. Similarly, the study further propels the community’s recommended green measures should be considered to plan risk-reducing contingencies against floods and heat island effects. Such bottom-up green initiatives promote community stewardship in green space planning, improve cities’ ecological resilience, and benefit dwellers in times of climate emergencies. These measures need immediate attention by the concerned stakeholders for the mitigative measure, followed by other inclusion of additional UGS elements in the landscape greening policies and planning for adaptive measures. All in all, the findings contribute to achieving an agreement on establishing a sustainable and inclusive UGI framework backed by the community’s understanding/importance. This may lead to building a new regional paradigm, which would encourage green growth infrastructure, not only in KP province but also in other regions having the same features.

It is worth mentioning that if this research outcome is compared with the native experts studied [34], it exemplifies both the native multi-stakeholder’s viewpoints on understanding the functional interlinkage between taxonomy of UGS elements and UGI indicators in the native built environment vary in some optimal possibilities. However, in most cases, the collective level of agreement overlapped/agreed. This reflects the knowledge, awareness, and perspective of native experts [34,51] and the community toward the natural green landscape-based (NBLB) approach, a sustainable, cost-efficient, and innovative climate change adaptation/mitigation approach for green cities. Additionally, the overall research studies represent a strong tendency to accentuate the holistic and effective multi-stakeholder participatory planning (MSPP) approach in the decision-making process for designing and implementing NBLG initiatives that naturally alleviate the high risk of environmental hazards in the northwest urban region of Khyber Pakhtunkhwa, Pakistan.

**Table 8:** Key Urban Green Space (UGS) elements.

Categories	Urban Green Infrastructure Indicators	Interquartile Range (IQR) Methdology				Cut-Off Point.	Approved Number of UGS Elements (RII ≥ 0.70)	Approved Urban Green Space (UGS) Elements
		Q1	Q3	IQR = (Q3-Q1) (Median)	Mean			
**Ecological**	**“Optimize storm water management”.**							
	i. “Increasing pervious surfaces”.	0.61	0.76	0.72	0.70	0.70	6	CG; BG; UP; FO; RG; WL
	ii. “Minimize, retain and organically-purified rainwater runoff”.	0.67	0.82	0.71	0.70	0.70	6	FO; GS; RG; GPA; WL; GRW.
	**“Decreasing the impact of urban heat islands”.**							
	iii. “Enhanced the quantity of the green spaces”.	0.59	0.72	0.66	0.70	0.70	4	CG; BG; UP; FO.
	iv. “Use of evaporative materials on the roofs, walls and floors”.	0.66	0.85	0.75	0.70	0.70	6	UP;FO;RG:GPA;WL; GRW
	**“Enhancing air quality (e.g., extracting impurities)”.**							
	v. “Growing more green trees and installing a green barrier in a roadway”.	0.60	0.69	0.67	0.70	0.70	6	CG; BG; UP; FO; GS; GRW.
	**“Enhancing noise quality”.**							
	vi. “Use a green sonic wall to reduce the minimum and maximum noise pollution. (i.e., thick hedges could be provided with a small meadow for minimum noise and for maximum noise reduction wide layers of bamboo and deciduous trees could be provided)”.	0.64	0.75	0.70	0.70	0.70	2	FO; UP.
	**“Lower emissions of carbon (e.g., elimination of greenhouse gas emissions through greenery)”**							
	vii. “Grow greater density of trees as shading and evaporating fabric for the paved surfaces”.	0.64	0.75	0.70	0.70	0.70	5	CG; BG; UP; FO; GS.
	**“Enhancing building energy performance”.**							
	viii. “Promote green energy-saving strategies”.	0.52	0.64	0.60	0.70	0.70	1	GRW
	**“Improved soil fertility and degradation condition”.**							
	ix. “Increase previous areas and plant trees to enhance soil stabilization”.	0.64	0.74	0.70	0.70	0.70	6	CG; BG; UP; FO; WL; HO.
	**“Improved and safeguard urban ecology”.**							
	x. “Improve and strengthen the urban green network connectivity”.	0.66	0.74	0.70	0.70	0.70	6	CG; BG; UP; FO; WL; HO.
**Socio-** **cultural**	**i. “Agri-production (e.g., home gardening; urban farming; and community farming)”.**	0.47	0.74	0.61	0.70	0.70	3	CP; FO; HO.
	**“Enhancing social wellness”**							
	ii. “Optimizing the recreation, and socialization activities”.	0.68	0.80	0.73	0.70	0.70	6	CG; BG; UP; FO; GS; GRW
	iii. “Improved city’s appeal (through various green elements)”.	0.74	0.79	0.76	0.70	0.70	9	CG; BG; UP; FO; GS; RG; GRW; WL; HO.
	**iv. “Enhancing the mental and physical health (e.g., visual and physical exposure to open green areas has a beneficial effect on stress and anxiety reduction)”.**	0.63	0.78	0.75	0.70	0.70	6	CG; BG; UP; FO; GR; WL
	**v. “Provide ecological areas for research & education”.**	0.67	0.76	0.72	0.70	0.70	6	CG; BG; FO; WL; GRW; HO
	**vi. “Enhance connectivity of green areas to promote walking & biking opportunities”.**	0.56	0.78	0.70	0.70	0.70	5	BG; FO; UP; GS; WL.
**Economic** **indicators**	**i. “Enhanced the value of property”.**	0.58	0.74	0.72	0.70	0.70	6	CG; BG; UP; GS; GRW; HO.
	**ii. “Minimize healthcare expense”.**	0.67	0.79	0.72	0.70	0.70	6	CG; BG; UP; FO; GS; GRW.
	**iii. “Decrease energy use (e.g., heating & cooling requirements)”.**	0.57	0.74	0.67	0.70	0.70	3	FO; UP; GRWl.
	**iv. “Minimize the risk of flood disasters”.**	0.66	0.74	0.72	0.70	0.70	7	CG; BG; UP; FO; GS; RG; WL.
	**v. “Decreasing the utilization of private cars by encouraging walking and biking opportunities (i.e., changing modes of transportation)”.**	0.53	0.78	0.69	0.70	0.70	5	BG; FO; UP; GS; WL.
	**vi. “Value of eliminating of air pollutants”.**	0.65	0.78	0.74	0.70	0.70	6	CG; BG; UP; FO; GS; GRW.

Source: Authors’ calculation using field survey data Keys: CG: “community garden”; BG: “botanical garden”; UP: “urban park”; FO: “forest”; GS: “green streets”; RG: “rain garden and bio-swale”; GPA: “green permeable parking area”; WL: “wetland”; GRW: “green roofs and walls”.

## 4. Discussion

This research contributes to building up an inclusive and sustainable UGI framework, thereby connecting the local community (and their perspective) with the multi-functional urban green areas. Such an ecological interaction between humans and nature helps to understand NBGI techniques that reduce environmental hazards and promote urban sustainability [84]. This study also attempts to find UGI indicators, referred to as UGS elements, according to the local built-in context that remains vital to enhancing urban planning. This research establishes that (based on the spatial context), each UGS element has a distinctive characteristic that plays a unique role in improving the quality of respective UGI indicators to fight climatic disasters (e.g., urban floods, drought, etc.). Additionally, the cohabitation of diverse vital taxonomy of green elements and UGI indicators can lead to developing a sustainable UGI framework, which is relevant to the local built environment. It also leads to accomplishing (nature-based) green policies to adapt to climate change through resilient land-use planning [15,85,86]), whereas such green planning approaches further naturally minimize the high risk of urban flooding [23,45,87,88] and build long-term climate-resilient environment. Therefore, developing such resilient strategies remains crucial in areas that are not only highly vulnerable to in-daunting climatic challenges [3,89] but also remain susceptible due to the geographical location, hence requiring a reactive planning system [25,36] in a situation where the expansion of urban functions remains escalated [90,91]. 

The harsh regional realities continue to put pressure on the land cover and, thereupon, the decline of urban green-spaces [35]. Thus, the regions required adequate and effective urban landscape and greening policies (ULGP). The upgradation of the existent policies and initiation of new urban plans needs to be tapped local community perspective. Such an approach is considered more effective in apprehending the intricacy of human and ecosystem interactions [38,39,51,52]. It stands crucial to identify the vital taxonomy of UGS elements. These elements will have the potential to identify the key/reliable/sustainable UGI indicators according to the native built-in environment. 

This integration of the local concepts can build a consensus toward an integrated urban landscape and green infrastructure. It is built on the idea of stimulating community participation while considering them important stakeholders in executing the planning/process, though it is not much institutionalized and practiced yet [36,49,92]. So, this study endorses a communal approach to building a UGI indicators’-based model. Only such a model can contribute to building a green, climate-resilient city-state. This approach can better address ecological, socio-cultural, and economic issues in land use. This model facilitates building an eco-regional paradigm that supports the successful transition of green action plans (GAP) and serves the community more effectively at the grassroots level in the urban interface of the Peshawar, Mardan, and Charsadda districts of KP and beyond. 

## 5. Conclusions

The empirical study has outlined an explicit quantitative research methodology for developing a rich body of multi-functional UGI-indicator-based framework/model grounded upon TBL sustainability. This scientific UGI model is backed by the local community’s perspective, and it presents the significance and practicability of UGI indicators and the UGS elements as per the local built-in environment. The results exhibit that ten UGI indicators fall into the categories of “IMP” and the other eleven as “M-IMP”, whereas only one indicator received the “E-IMP” level. Furthermore, a varied catalog of vital UGS elements (for UGI indicators) was presented, subject to the building spatial context of the KP region. This depicts community insight and satisfaction level towards the respective green spaces and their relationship with each UGI indicator while coping with climatic hazards. Moreover, this study has emphasized the role of the local inhabitants in establishing a sustainable UGI framework, meeting the standards of a green, climate-resilient city in the north-western region of Pakistan. The participatory planning (PP) approach is recognized as the best tool that effectively promotes and strengthens community stewardship in the planning process for urban green spaces at the grassroots level. All in all, this research study bridges the planning gap and improves collaboration processes among the local inhabitants and relevant government institutions. It is to overcome the gap between technical knowledge and expertise in NBGI to achieve resilience in land-use planning. It will build local capacity to fight climate uncertainties more cost-effectively than the traditional grey infrastructure.

In conclusion, these empirical findings highlight the role of native community members in developing a sustainable UGI-indicator-based framework/model according to socio-cultural and ecological contexts. This will lead to building an eco-friendly and (green) climate-resilient city-state, not only specific to the northwest urban regions of KP province (Pakistan) but having its application to other regions. The research will inevitably open up a new area to study the potential role of innovative and indigenous NBGI initiatives in addressing sustainable climate-risk management (SCRM) strategies according to the native spatial environment. This planning technique can pave the way to meeting the goal of a well-balanced relationship between anthropocentrism and eco-centrism activities, not only specific to the urban interface of the KP region but also across the country.

### Policy Implications

This research proposes essential policies guidelines/changes to create resilient urbanism against climatic risks (such as urban flooding):

(1). Increase awareness and understanding among all the native inhabitants toward a better understanding of UGI planning, a sustainable, cost-efficient, and innovative nature-based climate adaptation strategy for spongy green cities.
(2). A need to develop an inclusive policy that supports community participation at all levels, which will then promote community ownership and further strengthens the planning process for UGS.
(3). Balanced, proactive planning reforms are essential that encourage collaborations among the decision-makers and the local community. It should be linked with bridging the planning gap and improving the scientific knowledge regarding green initiatives, extending from policy making to decision making and implementation for greener growth.
(4). Considering the UGI planning examples of the Netherlands and Germany, there is a high need to incentivize green grass-root initiatives that would foster eco-friendly living practices and local stewardship of green practices to build a sustainable environment.

## 6. Scope of Future Research

(1). Further research can be conducted to study the relationship of the same (and additional) variables across socio-demographic groups to design micro-level urban greening policies.
(2). The social dimension of the sustainable urban landscape and greening policies (ULGP) and frameworks at the macro, micro, and meso levels needs to be investigated that can help build a new cultural paradigm to support and monitor green urbanism.
(3). The scalability of urban green space (UGS) elements must coincide with the magnitude of the climate hazards, knowing the appropriate green)/natural-based climate mitigation and adaptation measures to plan safer, healthier, and climate-resilient urban regions.
(4). In studying and analyzing green spaces, it would be interesting to consider different species of green roofs in different climates. It will help to better understand the potential role of green roofs in reducing climatic stress and improving the ecosystems functions (ESF) and health/well-being of inhabitants. Green roofs are becoming increasingly popular, especially in high-density urban clusters, where open spaces are limited. It is easy to implement and monitor, and they offer similar benefits as traditional green spaces.
(5). Pandemics (such as COVID-19) though pose less stress and do not degrade the UGI indicator more exclusively; however, this aspect needs to be further explored. There is a need to develop institutional and political indicators, and their potential role in NBGI infrastructure planning to address SCRM should be investigated.

## Figures and Tables

**Table 1:** Population census of three districts of KP province.

District	Tehsil	Town	Population	Geographic Data	Climate	Precipitation (mm) (1999–2018)
Mardan	Mardan Tehsil		1,403,394	34.2883° N, 72.1890° E	Humid subtropical	400.3
	Katlang Tehsil		343,144	34.3521° N, 72.0764° E		
	Takht Bhai Tehsil		626,523	34.3314° N, 71.9046° E		
Charsadda	Charsadda Tehsil		804,194	34.2165° N, 71.7148° E		
	Shabqadar Tehsil		383,765	34.2186° N, 71.5546° E		
	Tangi Tehsil		428,239	34.3040° N, 71.6555° E		
Peshawar	Peshawar Tehsil ([71])	Town 1	759,595	33.9437° N, 71.6199° E		546.075
		Town 2	547,807			
		Town 3	821,059			
		Town 4	435,940			
		Peshawar cant.	70,741			

Source: Authors’ compilation from the KP Bureau of Statistics (2018) [66], KP Local Government [71] and Pakistan Metrological department (2019) [72].

**Table 2:** Sampling size for the community survey.

District	Tehsil *(Selection Grounded on a High Urban Population)*	Tehsil Population	Union Council Population *(Selection Grounded on a High Urban Population with the Integration of the Interquartile Range Technique (IQR)*	Total No of Sample Population (with 95 CI and + 5 MoE)	Average HH Size No *(Source: KP Bureau of Statistics)*	No of HHs Sample 399.6/6.2 399.5/7 339.7/5.6
Mardan	Mardan	1,403,394	411,148	399.6	6.2	64
Charsadda	Charsadda	804,194	350,483	399.5	7	57
Peshawar	Town3	821,059	575,409	399.7	5.6	71

Source: Authors’ own elaboration, compilation the [66].

**Table 3:** Socio-demographic analysis.

Socio-Demographics	Total Participants	Ratio
**Gender-specific**		
Male	126	65.6
Female	43	22.4
Diverse (the government of Pakistan recognizes the identification of “trans” as a third gender [80,81,82])	0	0
Prefer not to say	23	12
**Location**		
Charsadda	56	29.1
Mardan	46	24
Peshawar	67	34.9
Not mention	23	12
**Literacy**		
No education to elementary	0	0
Secondary education (SSC)	14	7.3
Intermediate	37	19.3
Higher education	141	73.4
Other (informal)	5	2.6
**Age**		
15–20 years	0	0
20–30 years	66	34.4
30–40 years	84	43.8
40–50 years	42	21.9
50–60 years	14	7.3

Source: Authors’ calculation, using field data.

**Table 4:** Criterion of 9-point scale.

1	Extremely unimportant	(e-unimp)	(0 ≤ RI < 0.2)
2	Moderately unimportant	(m-unimp)	(0.2 ≤ RI < 0.3)
3	Slightly unimportant	(s-unimp)	(0.3 ≤ RI < 0.4)
4	Unimportant	(unimp)	(0.4 ≤ RI < 0.5)
5	Low	(l)	(0.5 ≤ RI < 0.6)
6	Slightly important	(s-imp)	(0.6 ≤ RI < 0.7)
7	Moderately important	(m-imp)	(0.7 ≤ RI < 0.8)
8	Important	(imp)	(0.8 ≤ RI < 0.9)
9	Extremely important	(e-imp)	(0.9 ≤ RI ≤1)

Source: [34].

**Table 5:** Equation (1) A sample estimating the RII value of increasing pervious surfaces to optimize the stormwater management indicator.

RII = ΣW/(N × A) … (1)
W = Likert scale weights: assigned by participants to each indicator (1 to 9).
N = Total number of samples
A = The highest value on a Likert scale.
RII = (9 × 93) + (8 × 42) + (7 × 21) + (6 × 18) + (5 × 8) + (−4 × 3) + (−3 × 3) + (−2 × 2) + (−1 × 2)/(192 × 9) = 0.834
(as rated by a community member)

Source: Authors’ calculation using field survey data.

**Table 6:** RII value of sustainable UGI indicators.

Categories	Urban Green Infrastructure Indicators	Particpants (n)	Overall Weight	Relative Index RII = ΣW/(N × A)	Cut-Off Point.	Approved UGI Indicators (RII ≥ 0.80)	Rank Order Based on RII Value	Level of Signifance (9-Point Scale Criterion)
					Interquartile Range Technique (IQR)			
**Ecological**	**“Optimize storm water management”**							
	i. “Increasing pervious surfaces”	192	1441	0.834	0.80	yes	9	8
	ii. “Minimize, retain and organically purified rainwater runoff”.	192	1364	0.789	0.80	no	14	7
	**“Decreasing the impact of urban heat islands”**							
	iii. “Enhanced the quantity of the green spaces”.	192	1517	0.878	0.80	yes	3	8
	iv. “Use of evaporative materials on the roofs, walls and floors”.	192	1287	0.745	0.80	no	19	7
	**“Enhancing air quality (e.g., extracting impurities)”.**							
	v. “Growing more green trees and installing a green barrier in a roadway”.	192	1339	0.775	0.80	no	16	7
	**“Enhancing noise quality”.**							
	vi. “Use a green sonic wall to reduce the minimum and maximum noise pollution. (i.e., thick hedges could be provided with a small meadow for minimum noise and for maximum noise reduction wide layers of bamboo and deciduous trees could be provided)”.	192	1347	0.780	0.80	no	15	7
	**“Lower emissions of carbon (e.g., elimination of greenhouse gas emissions through greenery)”**							
	vii. “Grow greater density of trees as shading and evaporating fabric for the paved surfaces”.	192	1513	0.876	0.80	yes	4	8
	**“Enhancing building energy performance”.**							
	viii. “Promote green energy-saving strategies”.	192	1581	0.915	0.80	yes	1	9
	**“Improved soil fertility and degradation condition”.**							
	ix. “Increase previous areas and plant trees to enhance soil stabilization”.	192	1473	0.852	0.80	yes	6	8
	**“Improved and safeguard urban ecology”.**							
	x. “Improve and strengthen the urban green network connectivity”.	190	1430	0.836	0.80	yes	8	8
**Socio-** **cultural**	**i. “Agri-production (e.g., home gardening; urban farming; and community farming)”.**	192	1411	0.817	0.80	yes	10	8
	**“Enhancing social wellness”.**							
	ii. “Optimizing the recreation, and socialization activities”.	192	1402	0.811	0.80	yes	11	8
	iii. “Improved city’s appeal (through various green elements)”.	192	1275	0.738	0.80	no	21	7
	**iv. “Enhancing the mental and physical health (e.g., visual and physical exposure to open green areas has a beneficial effect on stress and anxiety reduction)”.**	192	1509	0.873	0.80	yes	5	8
	**v. “Provide ecological areas for research & education”.**	192	1304	0.755	0.80	no	18	7
	**vi. “Enhance connectivity of green areas to promote walking & biking opportunities”.**	192	1287	0.745	0.80	no	19	7
**Economic** **indicators**	**i. “Enhanced the value of property”.**	192	1244	0.720	0.80	no	22	7
	**ii. “Minimize healthcare expense”.**	192	1369	0.792	0.80	no	13	7
	**iii. “Decrease energy use (e.g., heating & cooling requirements)”.**	192	1448	0.838	0.80	yes	7	8
	**iv. “Minimize the risk of flood disasters”.**	192	1544	0.894	0.80	yes	2	8
	**v. “Decreasing the utilization of private cars by encouraging walking and biking opportunities (i.e., changing modes of transportation)”.**	192	1377	0.797	0.80	no	12	7
	**vi. “Value of eliminating of air pollutants”.**	192	1331	0.770	0.80	no	17	7

Source: Authors’ calculation using field survey data. Significance level keys: 1—extremely unimportant; 2—moderately unimportant; 3—slightly unimportant; 4—unimportant; 5—low; 6—slightly important; 7—moderately important; 8—important; 9—extremely important.

**Table 7:** RII values for each urban green space (UGS) element.

Categories	Urban Green Infrastructure Indicators	Relative Index (RII) of UGS Elements RII = ΣW/(N × A)									
		Community Garden	Botanical Garden	Urban Park	Forest	Green Streets	Rain Garden & Bio-swale	Green & Permeable Parking Area	Wetland	Green Roof & Green Wall	Horticultural
**Ecological**	**“Optimize storm water management”.**										
	i. “Increasing pervious surfaces”.	0.71	0.72	0.75	0.88	0.63	0.76	0.6	0.81	0.56	0.59
	ii. “Minimize, retain and organically-purified rainwater runoff”.	0.66	0.69	0.65	0.82	0.81	0.91	0.71	0.92	0.7	0.65
	**“Decreasing the impact of urban heat islands”.**										
	iii. “Enhanced the quantity of the green spaces”.	0.7	0.73	0.75	0.9	0.67	0.48	0.4	0.6	0.65	0.58
	iv. “Use of evaporative materials on the roofs, walls and floors”.	0.65	0.69	0.76	0.86	0.63	0.91	0.82	0.94	0.73	0.53
	**“Enhancing air quality (e.g., extracting impurities)”.**										
	v. “Growing more green trees and installing a green barrier in a roadway”.	0.72	0.73	0.79	0.84	0.74	0.55	0.58	0.65	0.71	0.63
	**“Enhancing noise quality”.**										
	vi. “Use a green sonic wall to reduce the minimum and maximum noise pollution. (i.e., thick hedges could be provided with a small meadow for minimum noise and for maximum noise reduction wide layers of bamboo and deciduous trees could be provided)”.	0.69	0.69	0.74	0.91	0.69	0.42	0.37	0.6	0.64	0.61
	**“Lower emissions of carbon (e.g., elimination of greenhouse gas emissions through greenery)”**										
	vii. “Grow greater density of trees as shading and evaporating fabric for the paved surfaces”.	0.73	0.76	0.76	0.91	0.72	0.41	0.41	0.63	0.67	0.65
	**“Enhancing building energy performance”.**										
	viii. “Promote green energy-saving strategies”.	0.63	0.57	0.64	0.68	0.64	0.38	0.34	0.51	0.87	0.55
	**“Improved soil fertility and degradation condition”.**										
	ix. ”Increase previous areas and plant trees to enhance soil stabilization”.	0.73	0.77	0.74	0.89	0.66	0.63	0.53	0.70	0.60	0.70
	**“Improved and safeguard urban ecology”.**										
	x. “Improve and strengthen the urban green network connectivity”.	0.71	0.79	0.75	0.93	0.65	0.42	0.44	0.70	0.68	0.70
**Socio-** **cultural**	**i. “Agri-production (e.g., home gardening; urban farming; and community farming)”.**	0.87	0.66	0.61	0.76	0.53	0.35	0.30	0.45	0.61	0.82
	**“Enhancing social wellness”**										
	ii. “Optimizing the recreation, and socialization activities”.	0.78	0.81	0.81	0.82	0.75	0.41	0.30	0.68	0.71	0.69
	iii. “Improved city’s appeal (through various green elements)”.	0.75	0.78	0.82	0.85	0.79	0.70	0.69	0.77	0.75	0.74
	**iv. “Enhancing the mental and physical health (e.g., visual and physical exposure to open green areas has a beneficial effect on stress and anxiety reduction)”.**	0.79	0.74	0.81	0.89	0.75	0.39	0.38	0.75	0.69	0.61
	**v. “Provide ecological areas for research & education”.**	0.72	0.79	0.68	0.85	0.67	0.45	0.42	0.74	0.72	0.76
	**vi. “Enhance connectivity of green areas to promote walking & biking opportunities”.**	0.69	0.76	0.83	0.89	0.78	0.35	0.35	0.71	0.55	0.58
**Economic** **indicators**	**i. “Enhanced the value of property”.**	0.74	0.74	0.85	0.63	0.74	0.51	0.52	0.56	0.82	0.70
	**ii. “Minimize healthcare expense”.**	0.82	0.77	0.80	0.88	0.73	0.42	0.34	0.67	0.70	0.68
	**iii. “Decrease energy use (e.g., heating & cooling requirements)”.**	0.69	0.68	0.75	0.75	0.66	0.45	0.33	0.55	0.90	0.61
	**iv. “Minimize the risk of flood disasters”.**	0.70	0.72	0.74	0.95	0.71	0.73	0.64	0.86	0.61	0.60
	**v. “Decreasing the utilization of private cars by encouraging walking and biking opportunities (i.e., changing modes of transportation)”.**	0.66	0.73	0.80	0.84	0.79	0.42	0.44	0.71	0.53	0.53
	**vi. “Value of eliminating of air pollutants”.**	0.72	0.78	0.79	0.92	0.75	0.43	0.45	0.64	0.76	0.69

Source: Authors’ calculation using field survey data.

## Data Availability

This manuscript contains all data produced or examined during this investigation.

## Appendix B

**Table A1:** List of concepts evolved from the semi-structured meetings with native experts.

Mitigation of climate change	Adaptation to climate change	Water management
Green space networks	Ecosystem functions and services	Wildlife and biodiversity
Urban resilience	Organic food production	Energy-efficient building
Social cohesion/unity	A green economy	

Source: [34].

## Appendix C

Based on the inputs provided by the participants in the pilot survey, some minor revisions were made, aimed to make the survey design more appropriate and time-efficient:

- “Section A: In the participant profile, a diverse category had also been incorporated into the question of gender class. In Pakistan, the government officially recognized “trans” as the third gender [80,81,82]”.
- “In section b three-point Likert scale was updated into a five-point, and in Section C five-point Likert scale was transformed into a nine-point, aimed to achieve more variability among the respondent inputs and precision in the results”.
- “To mitigate the ambiguity among the participant’s feedback, certain queries of section c were also re-phrased”.

Source: [34].

## Appendix D

Identifying and validating the perspective, knowledge, beliefs, attitudes, and preferences of the native communities regarding the proposed definitions and possibilities of the UGI, urban resilience, climate change (CC), and adaptations to CC.

The following four questions were presented in section B of the community-based empirical survey executed in the KP districts of Charsadda, Peshawar, and Mardan, defining the concepts mentioned above according to the native built-in environment.

- “What does climate change mean for you?”
- “What does adaptation to climate change mean for you?”
- “What does urban resilience mean for you?”
- “What does green infrastructure mean for you?”

Source: [34].
